# Supplementary material for: Structural library and visualization of endogenously oxidized phosphatidylcholines using mass spectrometry-based techniques
Source: Nat Commun. 2021 Nov 3;12:6339. doi: 10.1038/s41467-021-26633-w (PMC8566498; doi:10.1038/s41467-021-26633-w)
Supplement: Supplementary file 1 — Supplementary Information [file 41467_2021_26633_MOESM1_ESM.pdf]

Supplementary Information for

Structural library and visualization of endogenously oxidized phosphatidylcholines using mass spectrometry-based techniques

Yuta Matsuoka[1], Masatomo Takahashi[2], Yuki Sugiura[3], Yoshihiro Izumi[2], Kazuhiro Nishiyama[4], Motohiro Nishida[4, 5], Makoto Suematsu[3], Takeshi Bamba[2], and Ken-ichi Yamada[1]*

[1]Physical Chemistry for Life Science Laboratory, Faculty of Pharmaceutical Sciences, Kyushu University, 3-1-1 Maidashi Higashi-ku, Fukuoka 812-8582, Japan.

[2]Metabolomics Laboratory, Research Center for Transomics Medicine, Medical Institute of Bioregulation, Kyushu University, 3-1-1 Maidashi Higashi-ku, Fukuoka 812-8582, Japan.

[3]Department of Biochemistry, Keio University School of Medicine, 35 Shinanomachi, Shinjuku-ku, Tokyo 160-8582, Japan.

[4]Department of Physiology, Faculty of Pharmaceutical Sciences, Kyushu University, 3-1-1 Maidashi Higashi-ku, Fukuoka 812-8582, Japan.

[5]Division of Cardiocirculatory Signaling, National Institute for Physiological Sciences and Exploratory Research Center on Life and Living Systems, National Institutes of Natural Sciences, 5-1 Higashiyama, Myodaiji-cho, Okazaki 444-8787, Japan.

*Corresponding author. Tel.: +81-92-642-6624, Fax: +81-92-642-6626,
e-mail: kenyamada@phar.kyushu-u.ac.jp

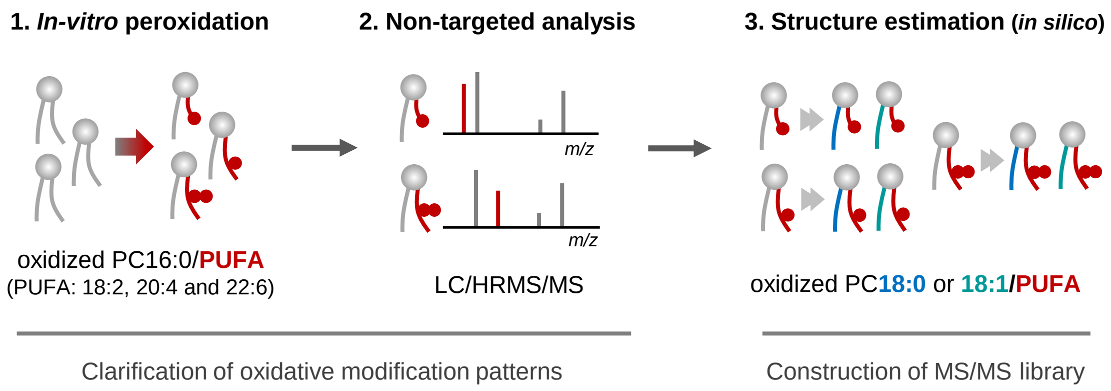

Supplementary Figure 1. Overview of oxPC library construction.

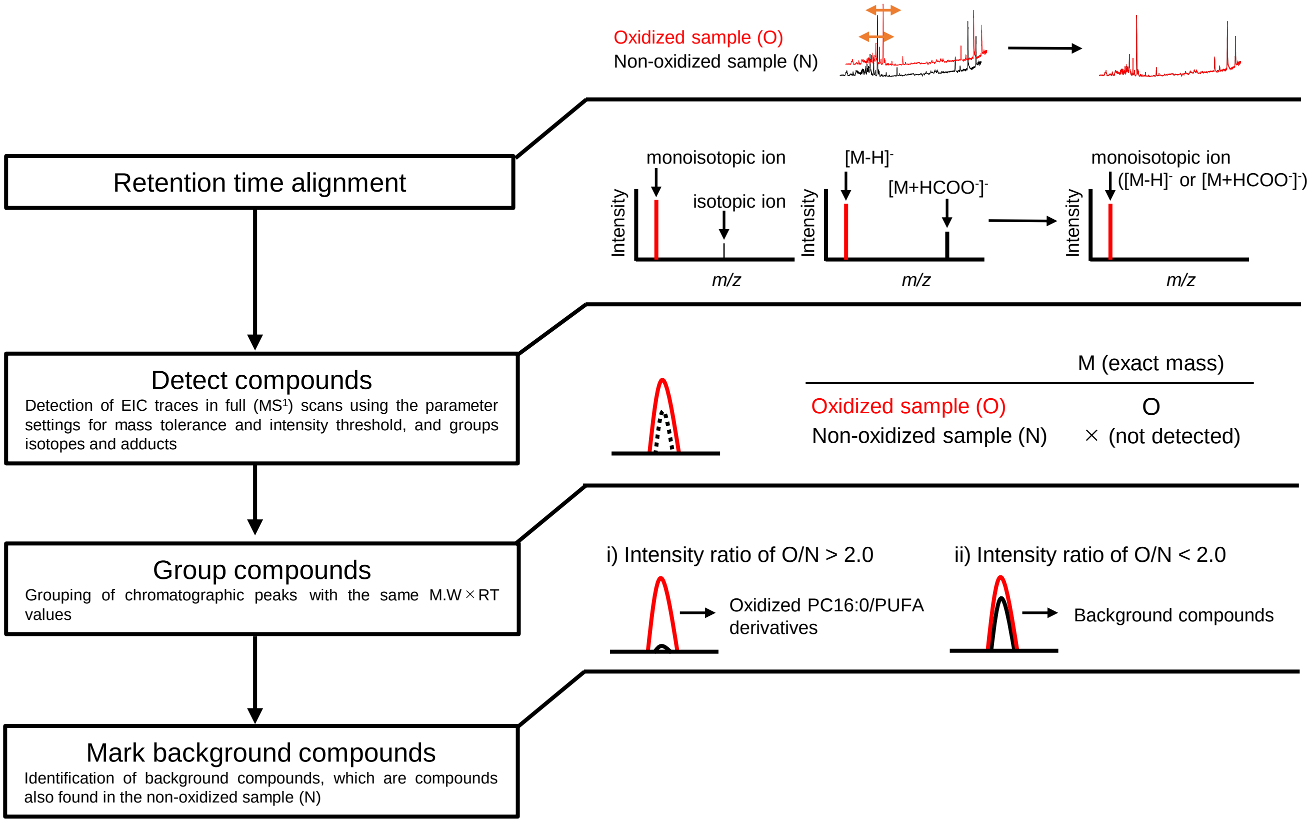

Supplementary Figure 2. Flowchart elucidating the steps involved in data processing using Compound Discoverer 3.1.

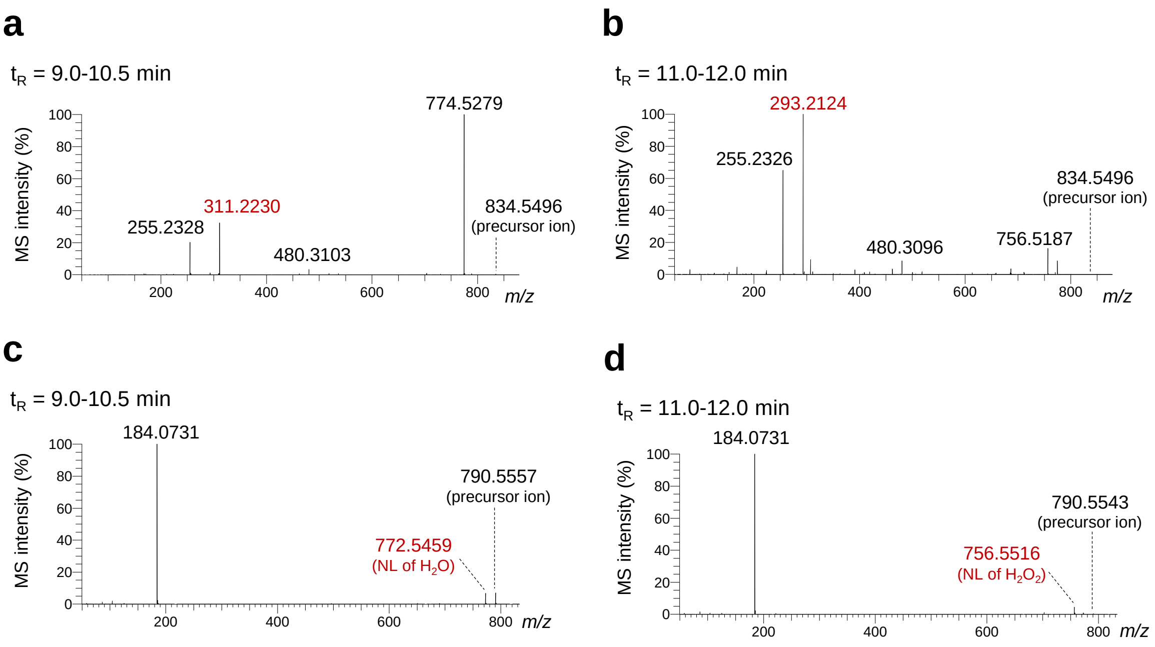

Supplementary Figure 3. HRMS/MS spectra of PC16:0_18:2;O2 in either negative or positive ion mode at t_R 9-10.5 and 11-12 min. a-b. HRMS/MS spectrum of *m/z* 834.5496 acquired under the negative ion mode at 9–10.5 (**a**) and 11–12 (**b**) min. **c-d.** HRMS/MS spectrum of *m/z* 790.5598 acquired under the positive ion mode at 9–10.5 (**c**) and 11–12 (**d**) min.

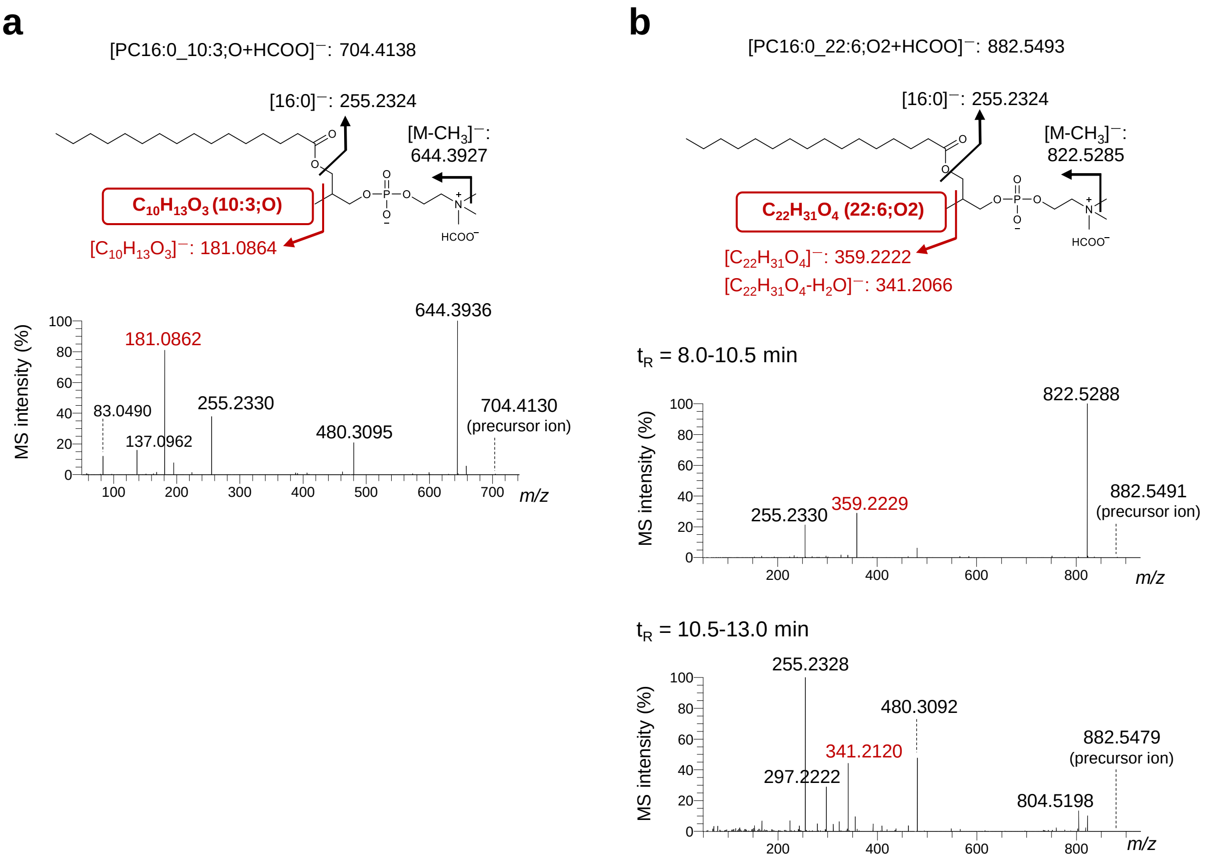

Supplementary Figure 4. Structural analysis of oxPCs derived from PC16:0/20:4 and PC16:0/22:6. a–b. HRMS/MS product ion spectra of PC16:0_10:3;O (*m/z* 704.4138, t_R = 7.0 min) (**a**) and PC16:0_22:6;O2 (*m/z* 882.5493, t_R = 8.0–10.5, 10.5–13.0 min) (**b**), which are derived from PC16:0/20:4 and PC16:0/22:6, respectively.

Supplementary Figure 5

a

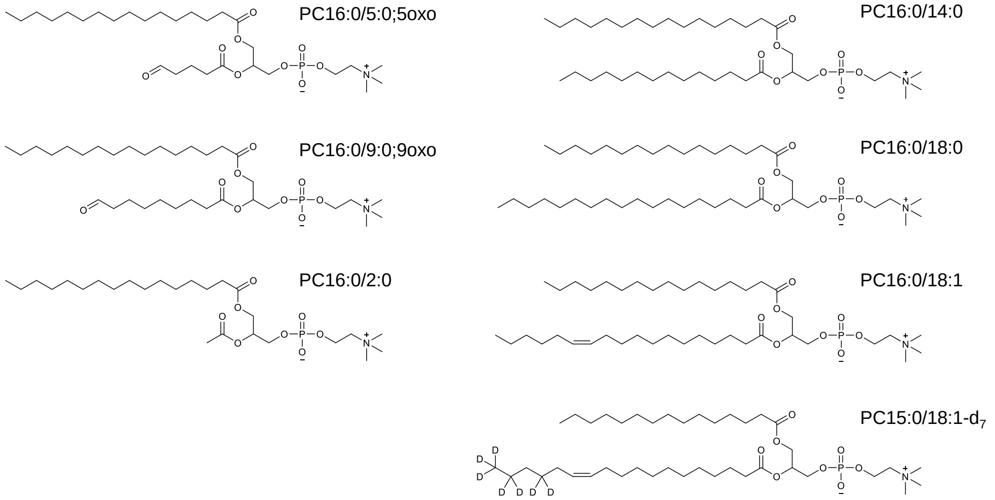

b

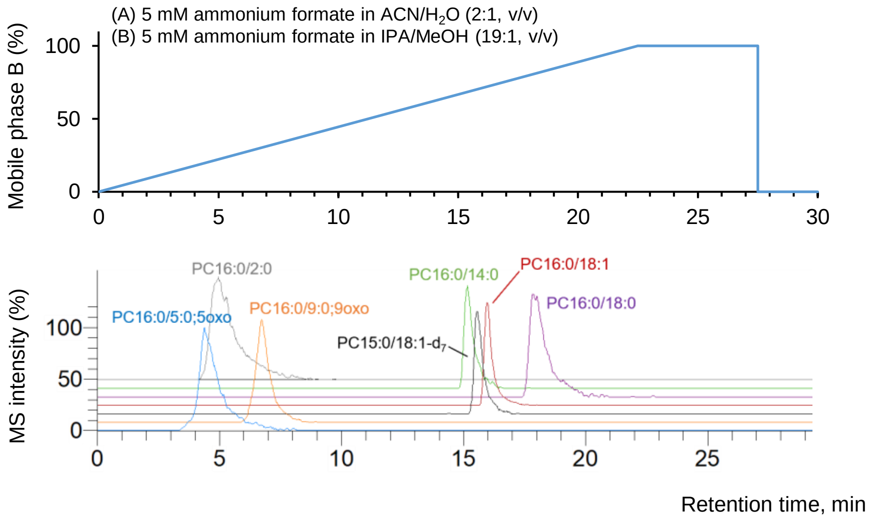

Continued Supplementary Figure 5

c

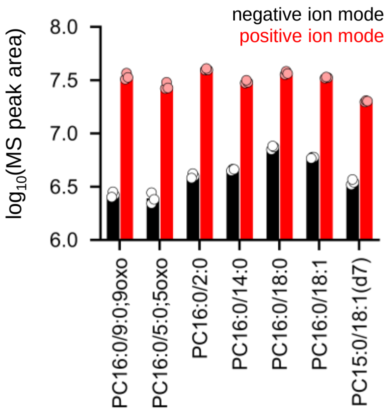

d

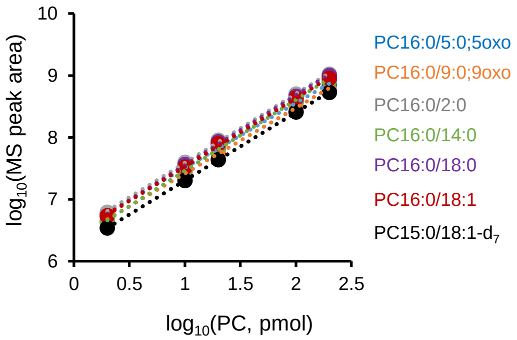

Supplementary Figure 5. LC/HRMS measurements of seven standard (ox)PCs. a. Chemical structures of the PCs employed in this experiment. **b.** HPLC gradient program and extracted-ion chromatograms (EICs) of 10 pmol standard (ox)PCs measured under the positive ion mode. **c.** LC/HRMS peak areas of each PC (10 pmol) measured under either negative (black) or positive (red) ion mode. LC/HRMS peak areas of PCs measured under the positive ion mode were higher than those measured under the negative ion mode. Data are presented as the mean + standard deviation of experiments repeated three times. **d.** Calibration curves for PC, pmol vs. LC/HRMS peak area measured under the positive ion mode. Data are presented as the mean ± standard deviation of experiments repeated three times. Source data are provided as a Source Data file.

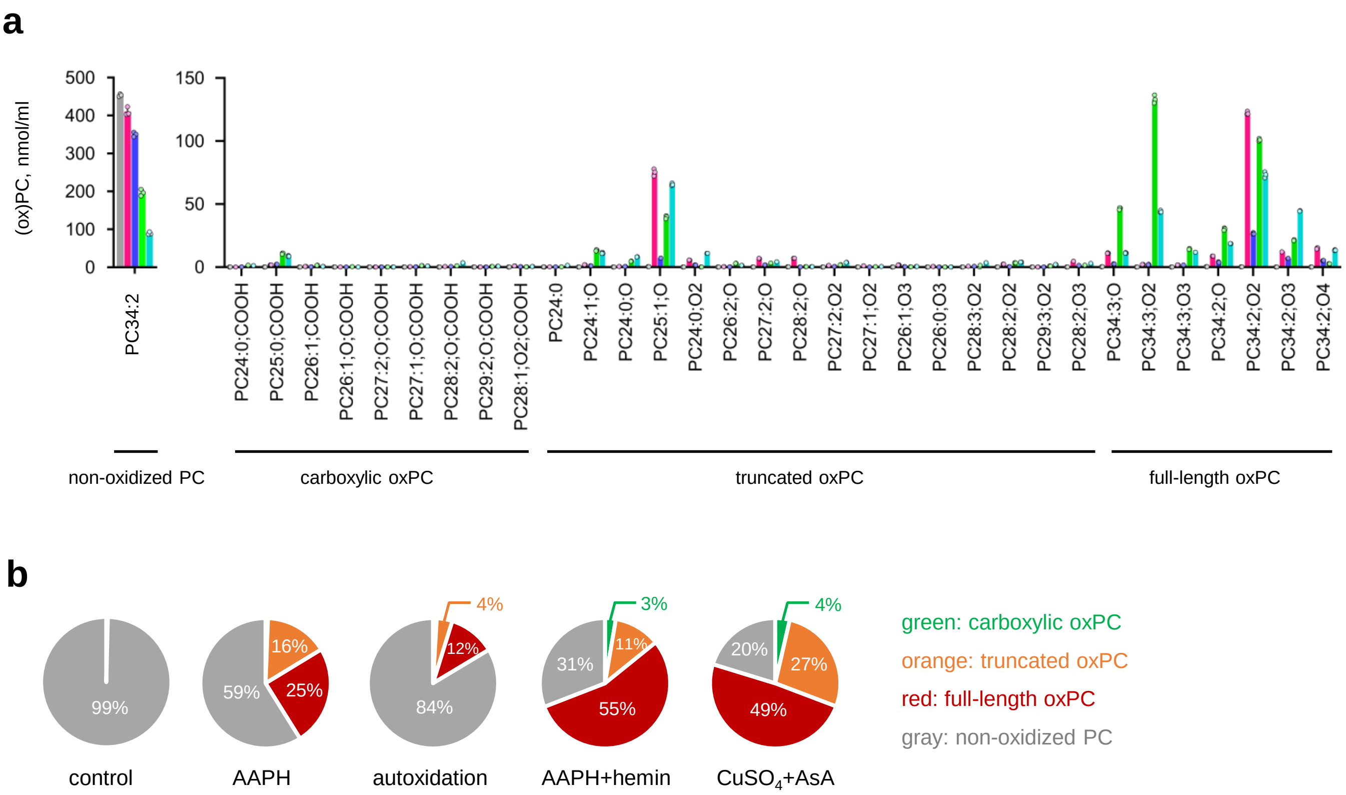

Supplementary Figure 6. Semiquantitative analysis of oxidized PC16:0/18:2 generated in several LPO systems. **a.** Semiquantified concentrations of non-oxidized and oxidized PC16:0/18:2 generated in different LPO systems (black, control; magenta, AAPH; blue, autoxidation; green, AAPH + hemin; and light blue, $CuSO_4$ + AsA). The peak areas from the extracted ion chromatogram (EIC) for each oxPC were determined by full-scan LC/HRMS in the positive ion mode. The semiquantitative values were calculated using the ratio of the MS peak area of individual oxPCs to that of PC15:0/18:1-d_7 (internal standard). Data are presented as the mean + standard deviation of experiments repeated three times. **b.** Proportions of non-oxidized (gray) or oxidized PCs (green, carboxylic oxPCs; orange, truncated oxPCs; red, full-length oxPCs) in the total amount of PCs. Source data are provided as a Source Data file.

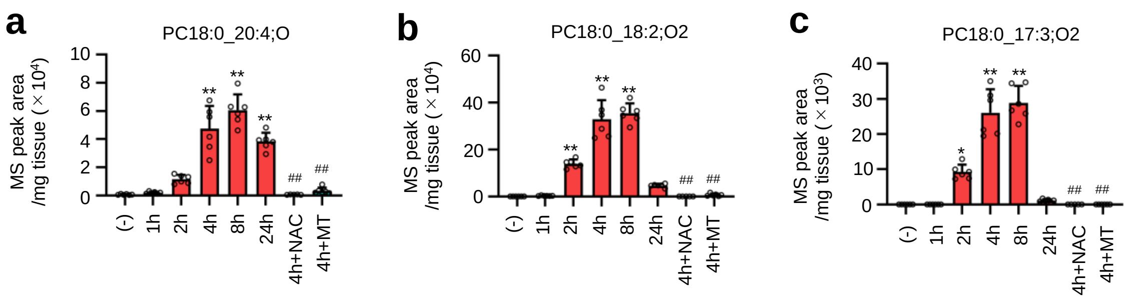

Supplementary Figure 7. Time-dependent differences in HRMS/MS peak areas obtained in the negative ion mode for PC18:0_oxPUFAs formed after APAP administration. Mouse liver samples were collected 1, 2, 4, 8, and 24 h after APAP (300 mg/kg in saline) administration. **a.** PC18:0_20:4;O, **b.** PC18:0_18:2;O2, and **c.** PC18:0_17:3;O2. LC/HRMS/MS data were obtained by using PRM in the negative ion mode. Data are presented as the mean + standard deviation of experiments repeated six times. *P* value was determined by the one-way ANOVA with the Tukey's multiple comparison test. **P* = 0.0001, ***P* < 0.0001, compared with the vehicle-treated group. ##*P* < 0.0001, compared with the APAP-treated group 4 h post-treatment. Source data are provided as a Source Data file.

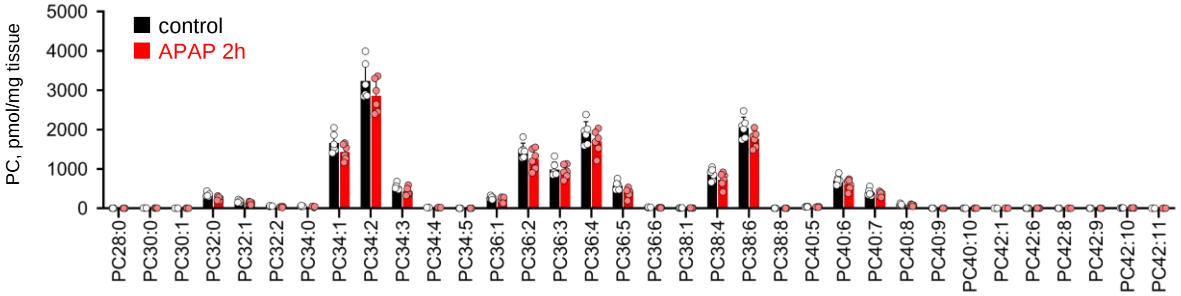

Supplementary Figure 8. Semiquantitative analysis of non-oxidized PCs in the mouse liver. Semiquantified concentrations of hepatic PCs in vehicle- (black) and 2-h APAP-treated (red) groups. The peak areas from the extracted ion chromatograms (EICs) for each oxPC were determined by full-scan LC/HRMS in the positive ion mode. The semiquantitative values were calculated using the ratio of the MS peak area of each oxPC to that of PC15:0/18:1-d7 (internal standard). Data are presented as the mean + standard deviation of experiments repeated six times. Source data are provided as a Source Data file.

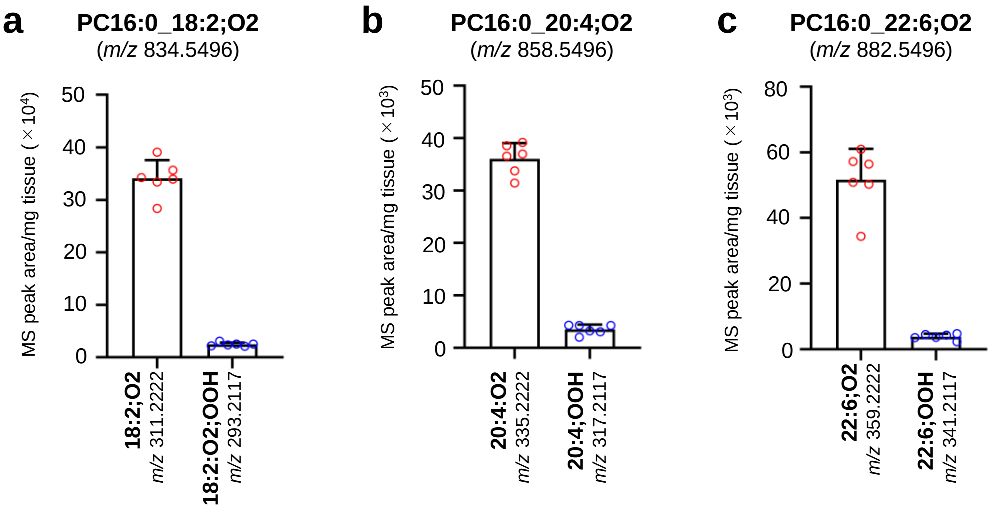

Supplementary Figure 9. Endogenously generated PC16:0_PUFA;O2 contains epoxide/hydroxide functionalities but not hydroperoxide functionality. a-c. Semiquantitative extracted ion chromatogram (EIC) peak areas of product ions corresponding to oxidized fatty acyls derived from PC16:0_PUFA;O2 (PC16:0_18:2;O2 (**a**), PC16:0_20:4;O2 (**b**), PC16:0_22:6;O2 (**c**)). Data are presented as the mean + standard deviation of experiments repeated six times. Source data are provided as a Source Data file.

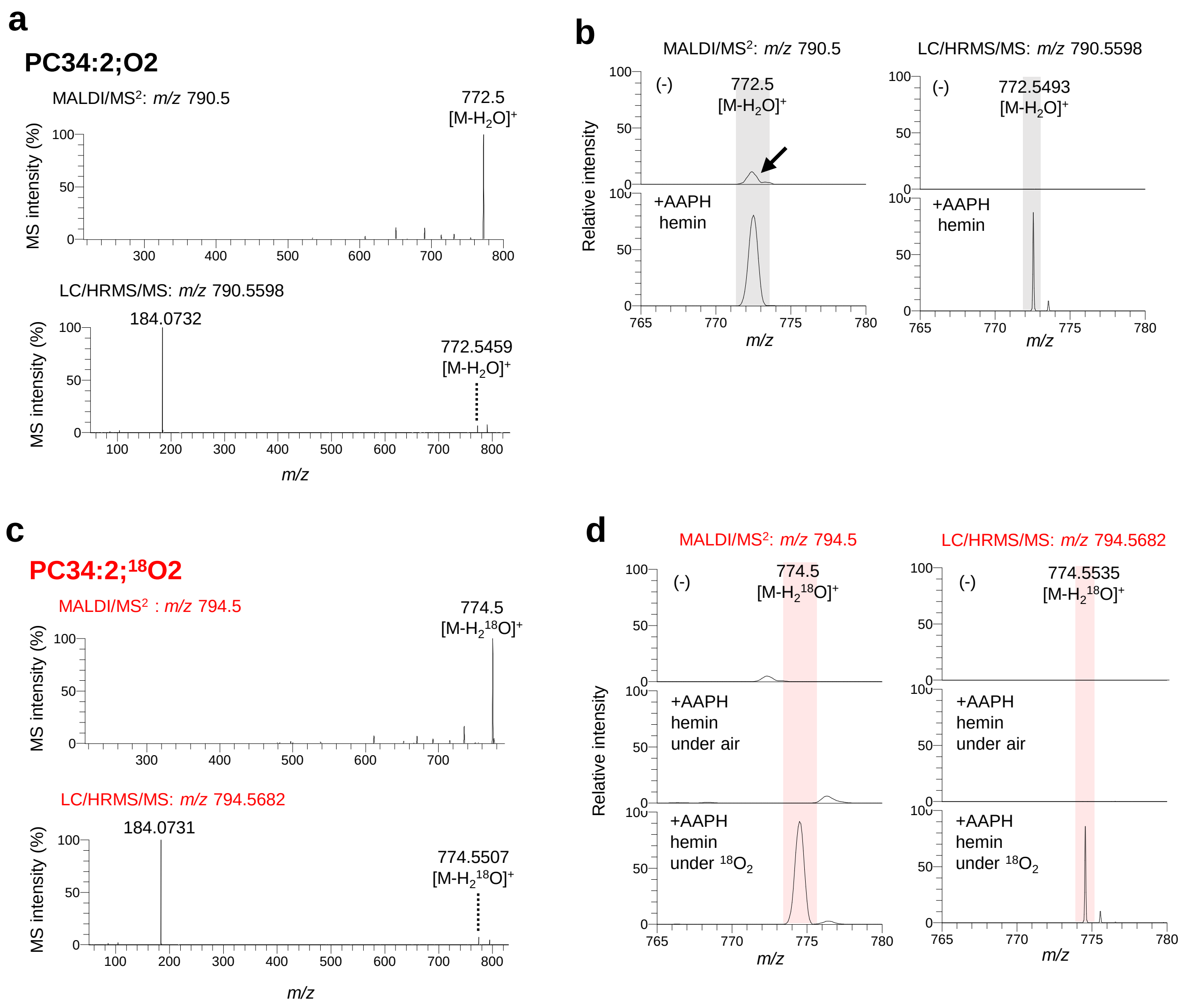

Supplementary Figure 10. MALDI/MS/MS and LC/HRMS/MS analyses of PC34:2;O2 and PC34:2;^{18}O2 generated by *in vitro* peroxidation. a, **c.** Product ion spectra of PC34:2;O2 (**a**) and PC34:2;^{18}O2 (**c**) obtained by either MALDI/MS/MS or LC/HRMS/MS analysis in the positive ion mode. To obtain PC34:2;O2 and PC34:2;^{18}O2, PC16:0/18:2 was oxidized using the AAPH + hemin peroxidation system in normal and $^{18}O_2$ air, respectively. Characteristic ions at *m/z* 772.5 and 774.5 correspond to the loss of H_2O and $H_2{}^{18}O$ from oxidized fatty acyl chains, respectively. **b**, **d.** MALDI/MS/MS and LC/HRMS/MS analyses of *m/z* 790.5 → 772.5 (**b**) and *m/z* 794.5 → 774.5 (**d**) transitions corresponding to the formation of PC34:2;O2 and PC34:2;^{18}O2, respectively. Samples were oxidized by AAPH + hemin treatment in normal or $^{18}O_2$ air.

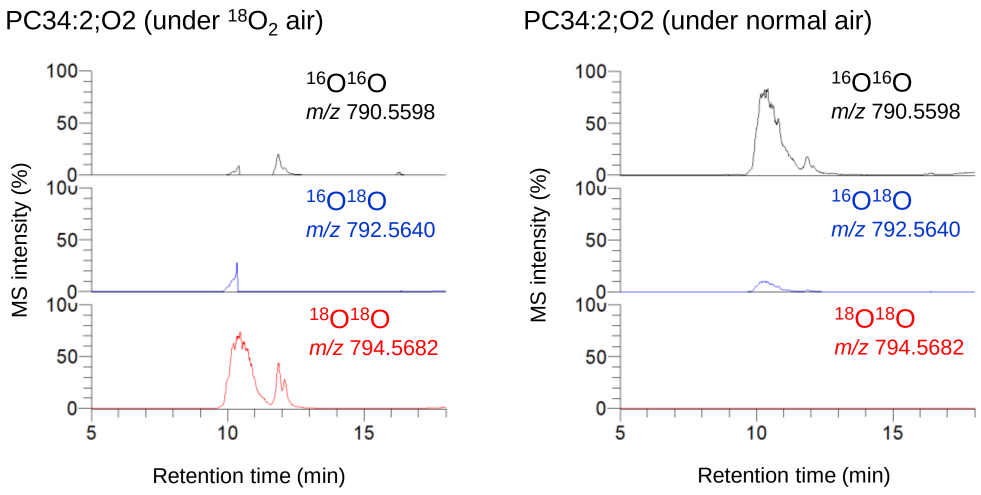

Supplementary Figure 11. ^{18}O conversion of PC34:2;O2 generated under AAPH + hemin peroxidation system in $^{18}O_2$ air. Extracted ion chromatograms (EICs) for ions with *m/z* 790.5598 (black), 792.5640 (blue), and 794.5682 (red) corresponding to PC34:2;$^{16}O_2$, PC34:2; $^{16}O^{18}O$, and PC34:2;^{18}O2, respectively. Left, right: PC34:2;O2 generated under AAPH + hemin peroxidation system in $^{18}O_2$ and normal air, respectively.

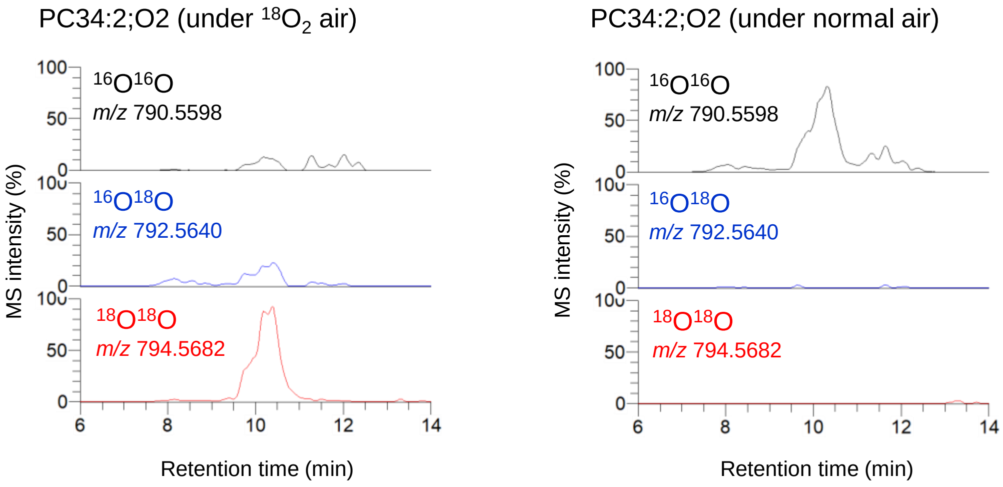

Supplementary Figure 12. ^{18}O conversion of PC34:2;O2 in APAP-treated mice in $^{18}O_2$ air. Extracted ion chromatograms (EICs) for ions with *m/z* 790.5598 (black), 792.5640 (blue), and 794.5682 (red) corresponding to PC34:2;$^{16}O_2$, PC34:2; $^{16}O^{18}O$, and PC34:2;^{18}O2, respectively. Left, right: APAP-treated groups kept for 2 h in $^{18}O_2$ and normal air, respectively.

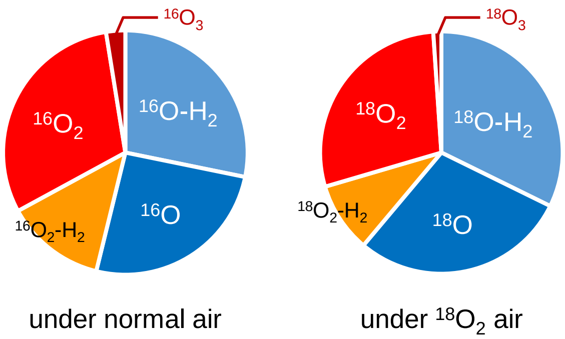

Supplementary Figure 13. Product profiles of oxidized PC16:0/18:2 generated in mice treated with APAP for 2 h and inhaling either normal or $^{18}O_2$ air. Source data are provided as a Source Data file.

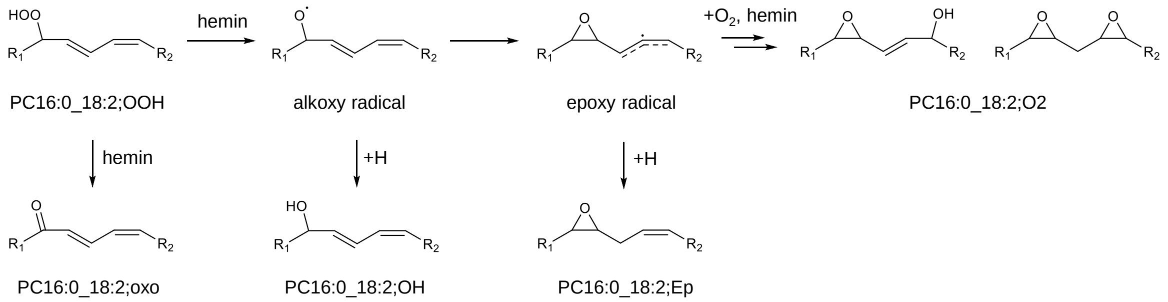

Supplementary Figure 14. Proposed mechanisms of formation of the secondary oxidation product (e.g., lipid ketone, hydroxide, and epoxide) via hemin-induced decomposition of lipid hydroperoxides.

HRMS/MS: *m/z* 838.5582; PC16:0_18:2;[18]O2

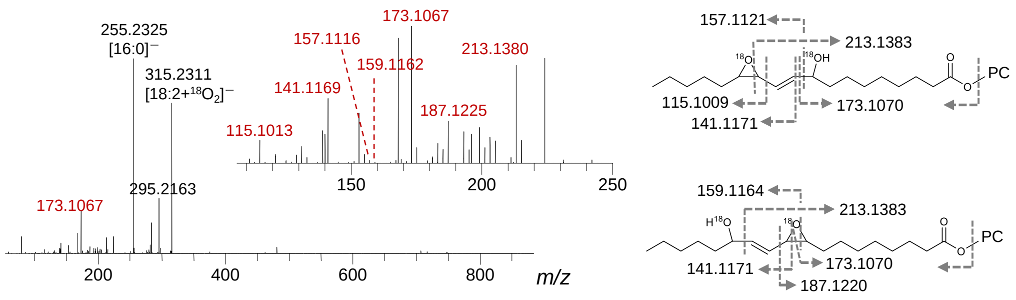

Supplementary Figure 15. LC/HRMS/MS identification and plausible structures of PC16:0_18:2;[18]O2 generated in mice treated for 2 h with APAP and inhaling $^{18}O_2$ air. HRMS/MS spectra of the product ion at *m/z* 838.5582 corresponding to PC16:0_18:2;[18]O2.

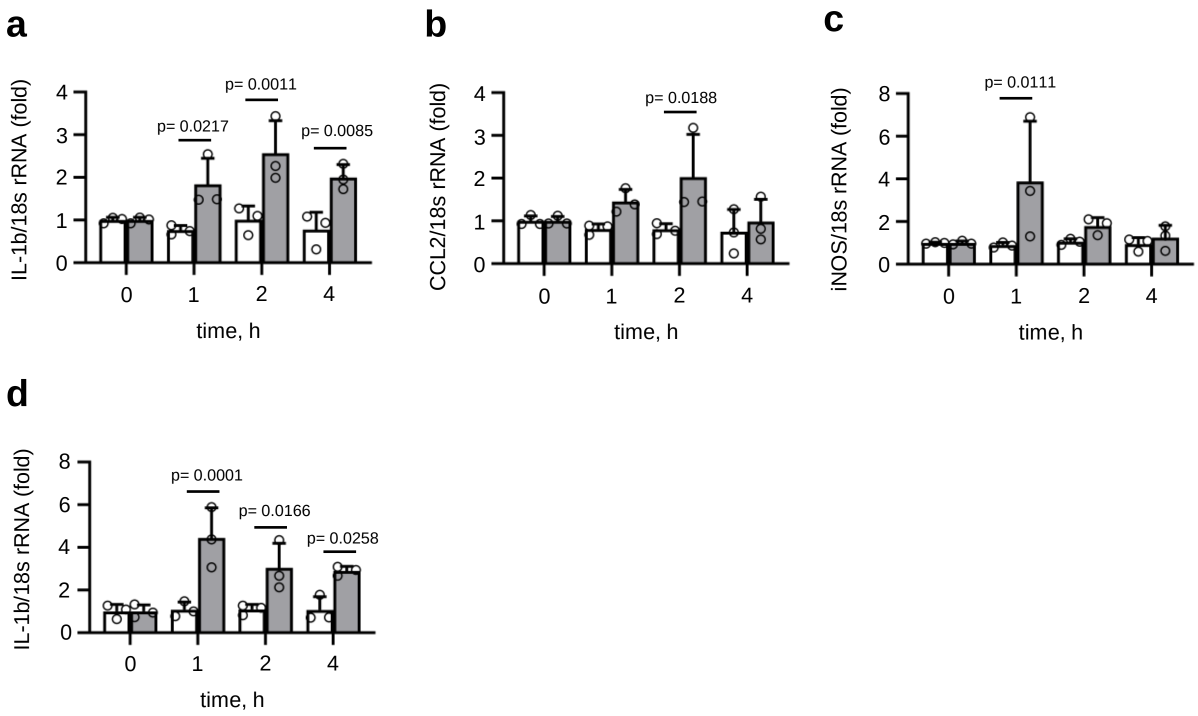

Supplementary Figure 16. Proinflammatory effects of oxidized PC16:0/20:4 and lipid extracts from mice livers on RAW 264.7 cells. a–c. RAW 264.7 cells were treated with 50 µM PC16:0/20:4 (white column) or oxidized PC16:0/20:4 (gray column) generated by AAPH + hemin peroxidation for 4 h. The mRNA expression levels of *Il-1b* (**a**), *Ccl2* (**b**), and *Nos2* (**c**) were analyzed at 1, 2, and 4 h post-treatment. Cells were homogenized, and total RNA was extracted using TRIzol reagent (Sigma-Aldrich, St. Louis, MO). cDNA was synthesized using the ReverTra Ace qPCR RT Master Mix (Toyobo, Japan) according to the manufacturer's instructions. Quantitative real-time PCR was performed using the ABI StepOnePlus Real Time System (Applied Biosystems) and KAPA SYBR FAST qPCR kit (Roche, Basel, Switzerland) according to the manufacturer's instructions. The following primer sequences were used for amplification: forward 5′-ATGGCAACTGTTCCTGAACTCAA-3′ and reverse 5′-CAGGACAGGTATAGATTCTTTCC-3′ (*Il-1b*), forward 5′ - TTAAAAACCTGGATCGGAACCAA - 3′ and reverse 5′ - GCATTAGCTTCAGATTTACGGGT - 3′ (*Ccl2*), forward 5′ - CCAAGCCCTCACCTACTTCC - 3′ and reverse 5′ - CTCTGAGGGCTGACACAAGG - 3′ (*Nos2*), and forward 5′ - ATTAATCAAGAACGAAAGTCGCAGGT - 3′ and reverse 5′ - TTTAAGTTTCAGCTTTGCAACCATACT - 3′ (18S rRNA). The PCR was set as follows: one cycle at 95 ° C for 10 min, followed by 40 cycles of 30 s at 94 ° C, 30 s at 60 ° C, and 10 s at 72 ° C. The relative fold-change in target gene expression was calculated using the $2^{-\Delta\Delta Ct}$ method as previously described.[1] 18S rRNA expression was used to normalize target gene expression. Data are presented as the mean + standard deviation of experiments repeated three times. Significance was determined using two-way ANOVA, followed by Sidak’s comparison test. **d.** RAW 264.7 cells were treated with lipid extracts from the livers of either normal (white) or 2-h APAP-treated (gray) mice. To obtain the extraction solutions, 200 mg mouse liver tissues were collected and hepatic lipids were extracted using the modified Bligh and Dyer method. The mRNA expression levels of Il-1b were analyzed at 1, 2, and 4 h post-treatment. Data are presented as the mean + standard deviation of experiments repeated three times. *P* value was determined using two-way ANOVA, followed by Sidak’s comparison test. Source data are provided as a Source Data file.

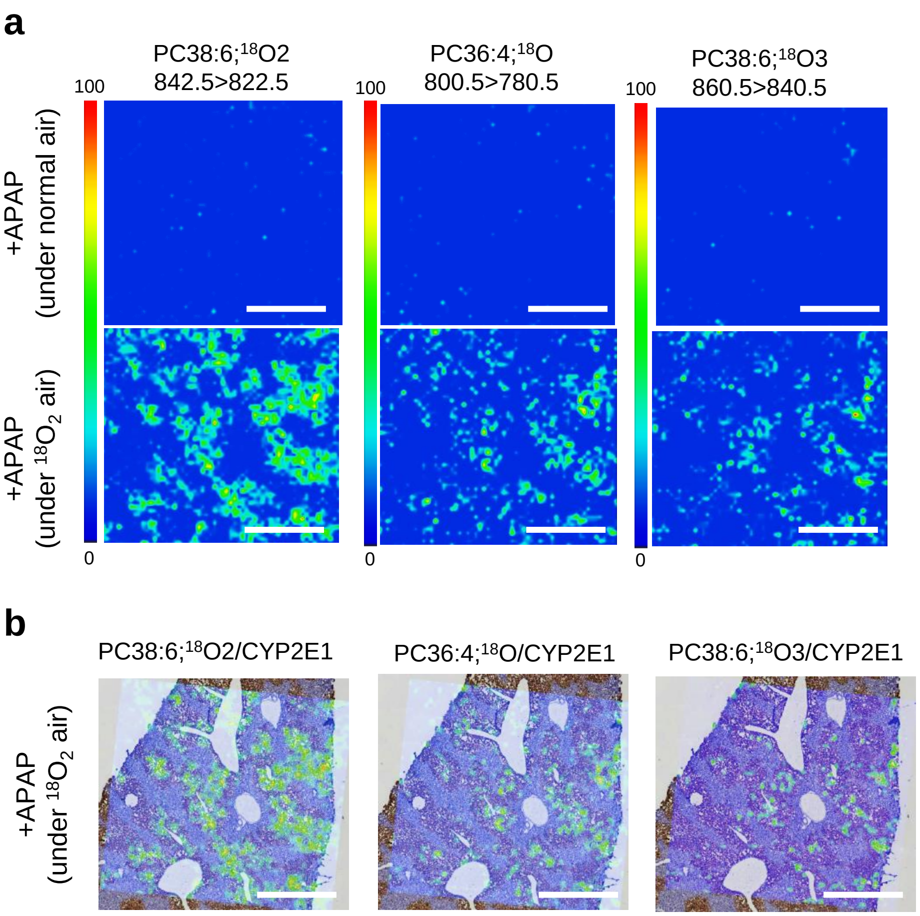

Supplementary Figure 17. Visualization of relatively abundant oxPCs, such as PC36:4;^{18}O and PC38:6;^{18}O3, by MALDI-MS/MS/MSI. a. PC36:4;^{18}O and PC38:6;^{18}O3 are observed only in APAP-treated mice inhaling $^{18}O_2$ air. In the pseudo color scale, the maximum intensity of the detected signal is set to "100" and the intensity 0 is set to "0". **b.** The localization of PC36:4;^{18}O and PC38:6;^{18}O3 matches well with the areas of CYP2E1 expression. Scale bar = 1 mm.

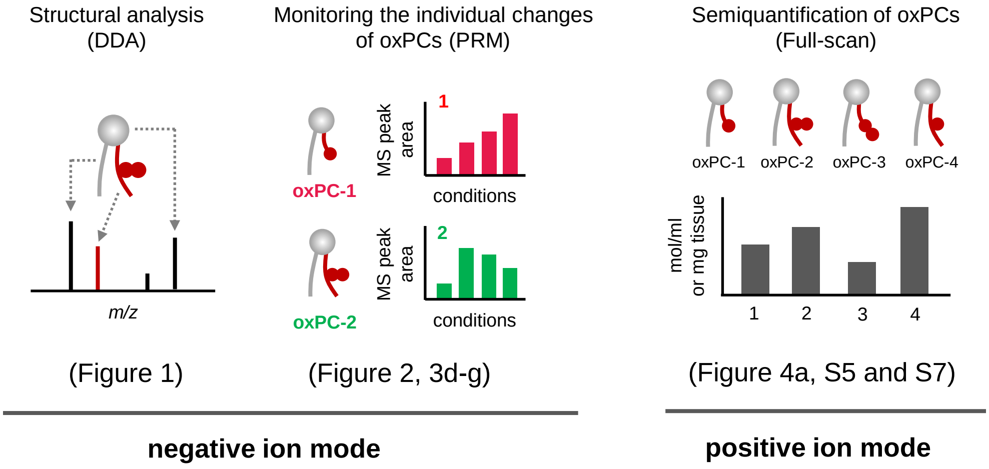

Supplementary Figure 18. Summary of the detection methods employed in this study. DDA; data dependent acquisition, PRM; parallel reaction monitoring.

Supplementary Figure 19

a

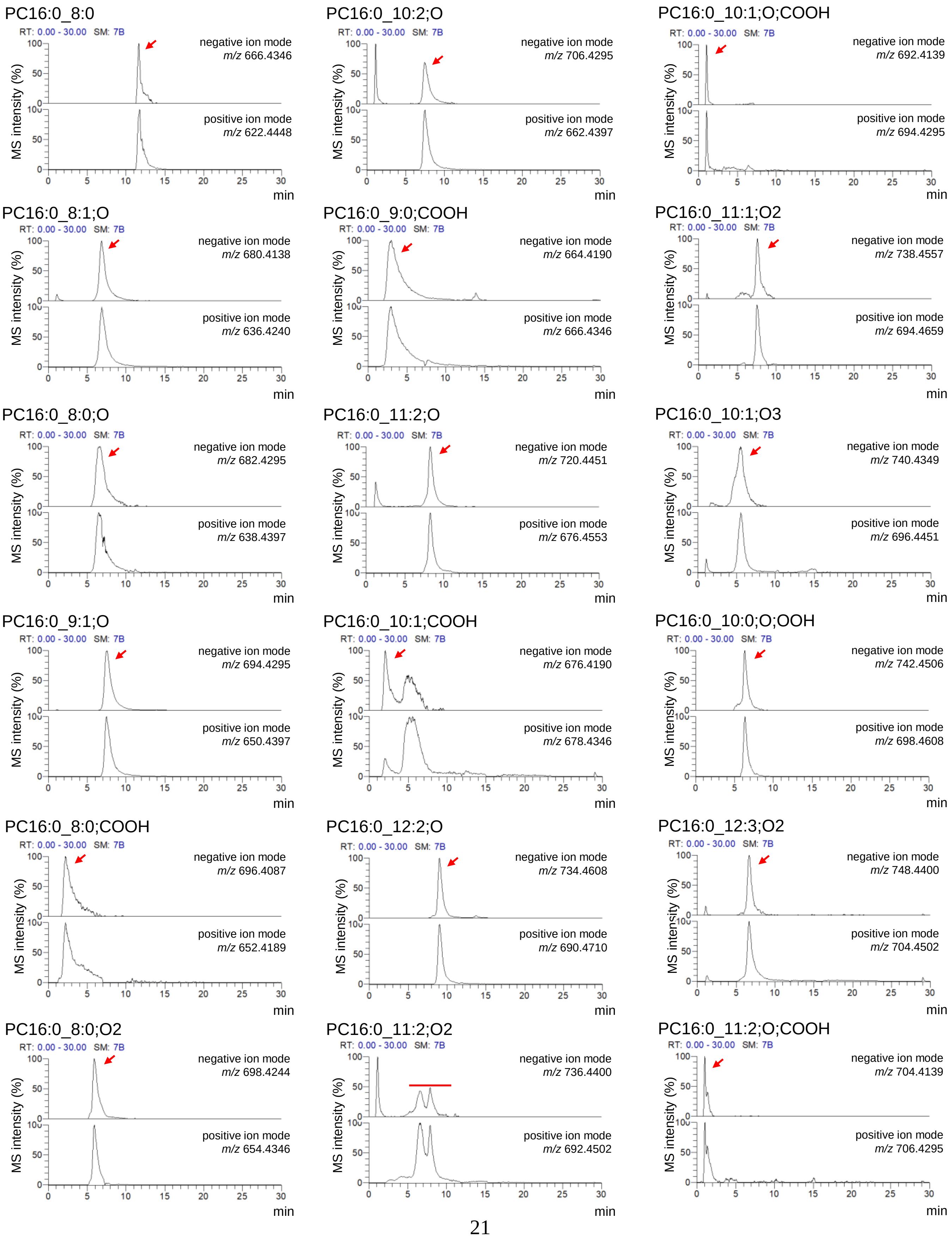

Continued Supplementary Figure 19

a

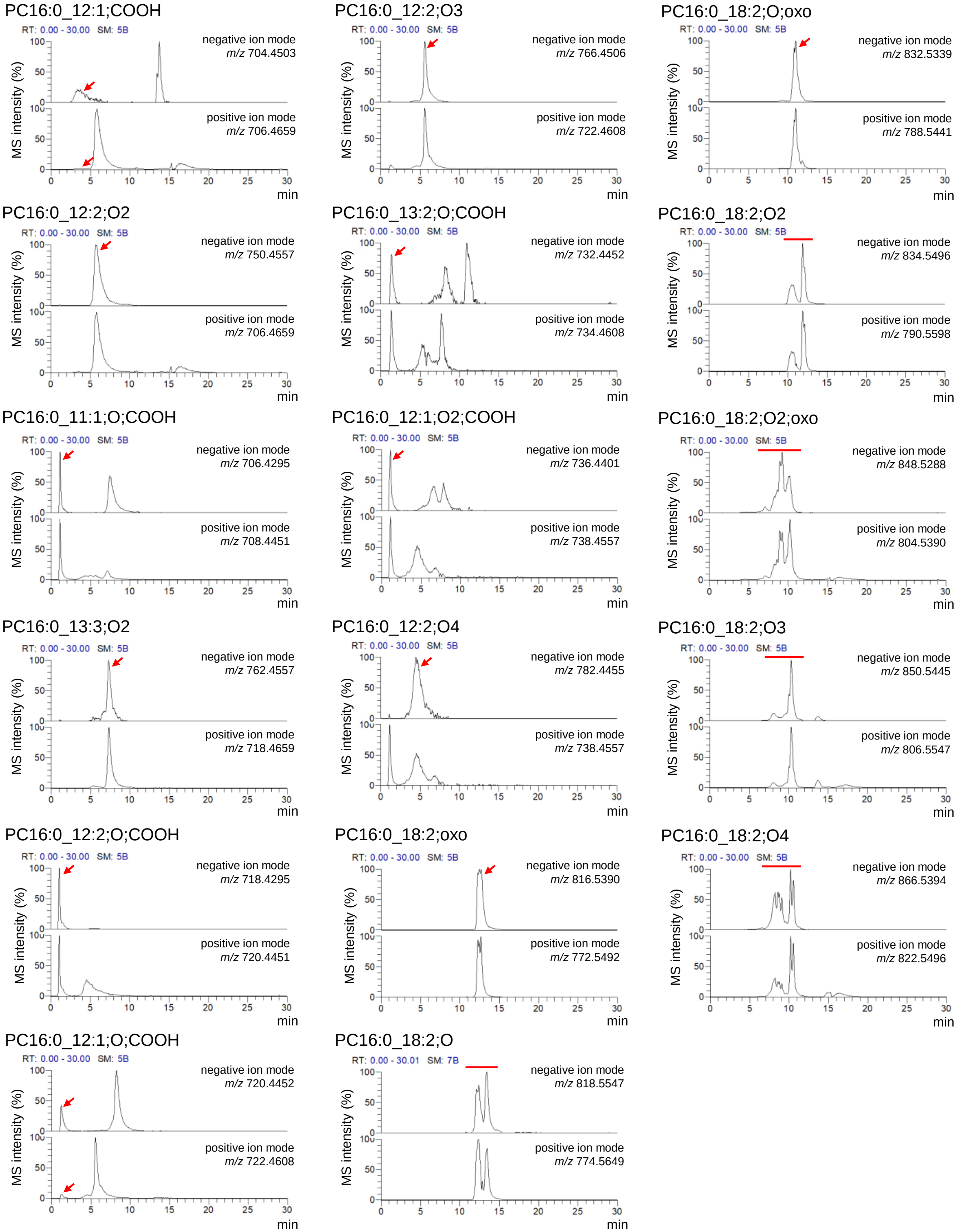

Continued Supplementary Figure 19

b

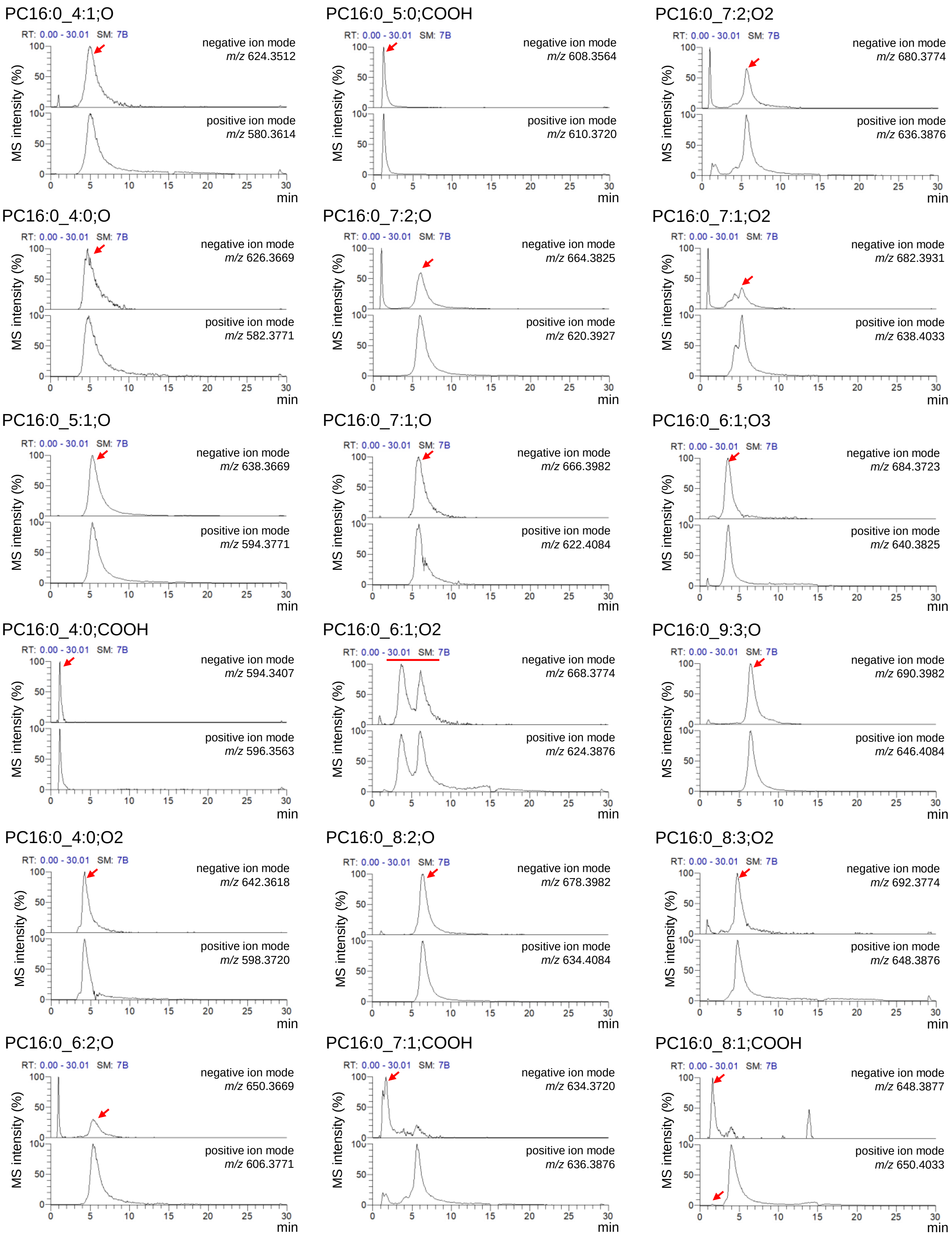

Continued Supplementary Figure 19

b

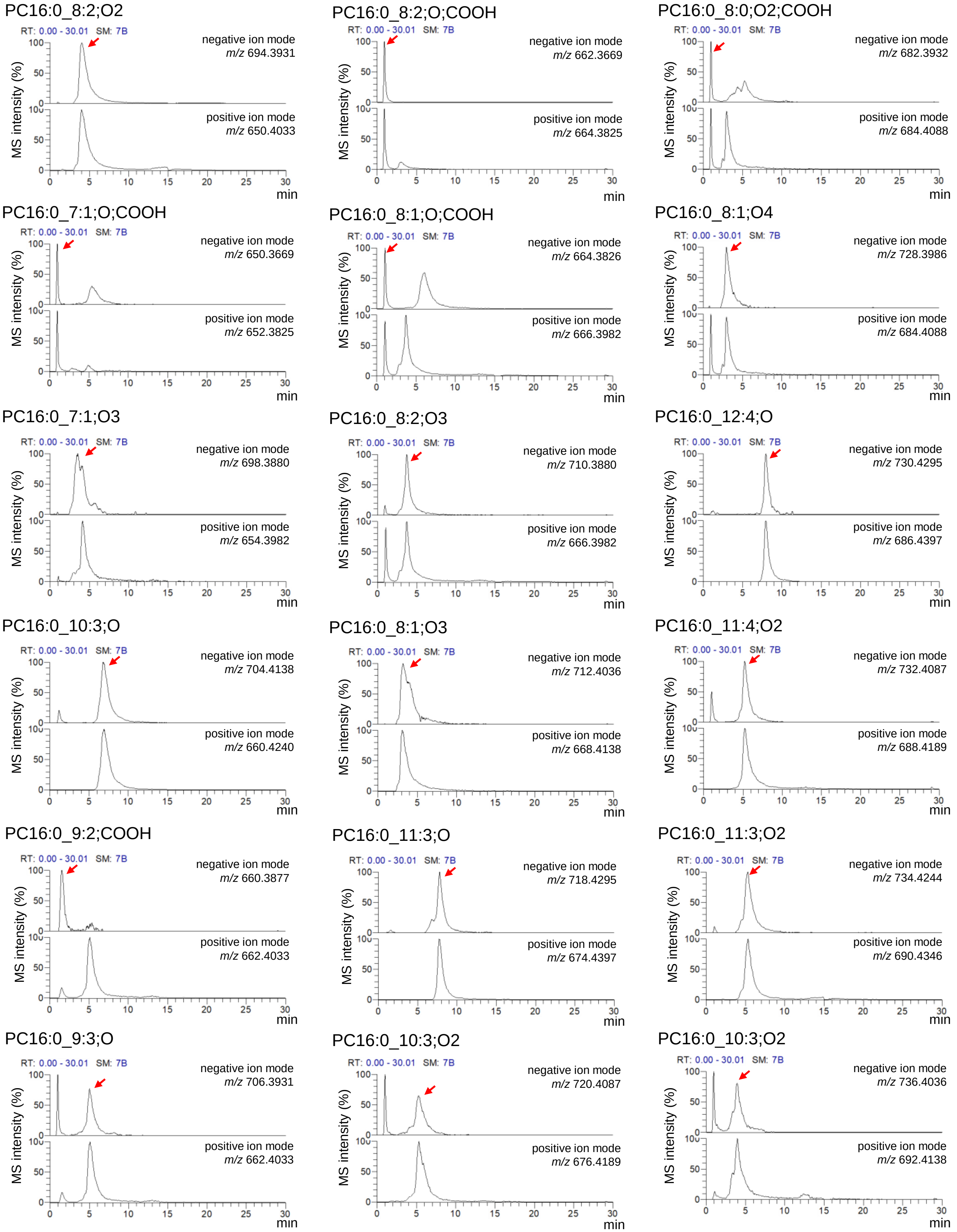

Continued Supplementary Figure 19

b

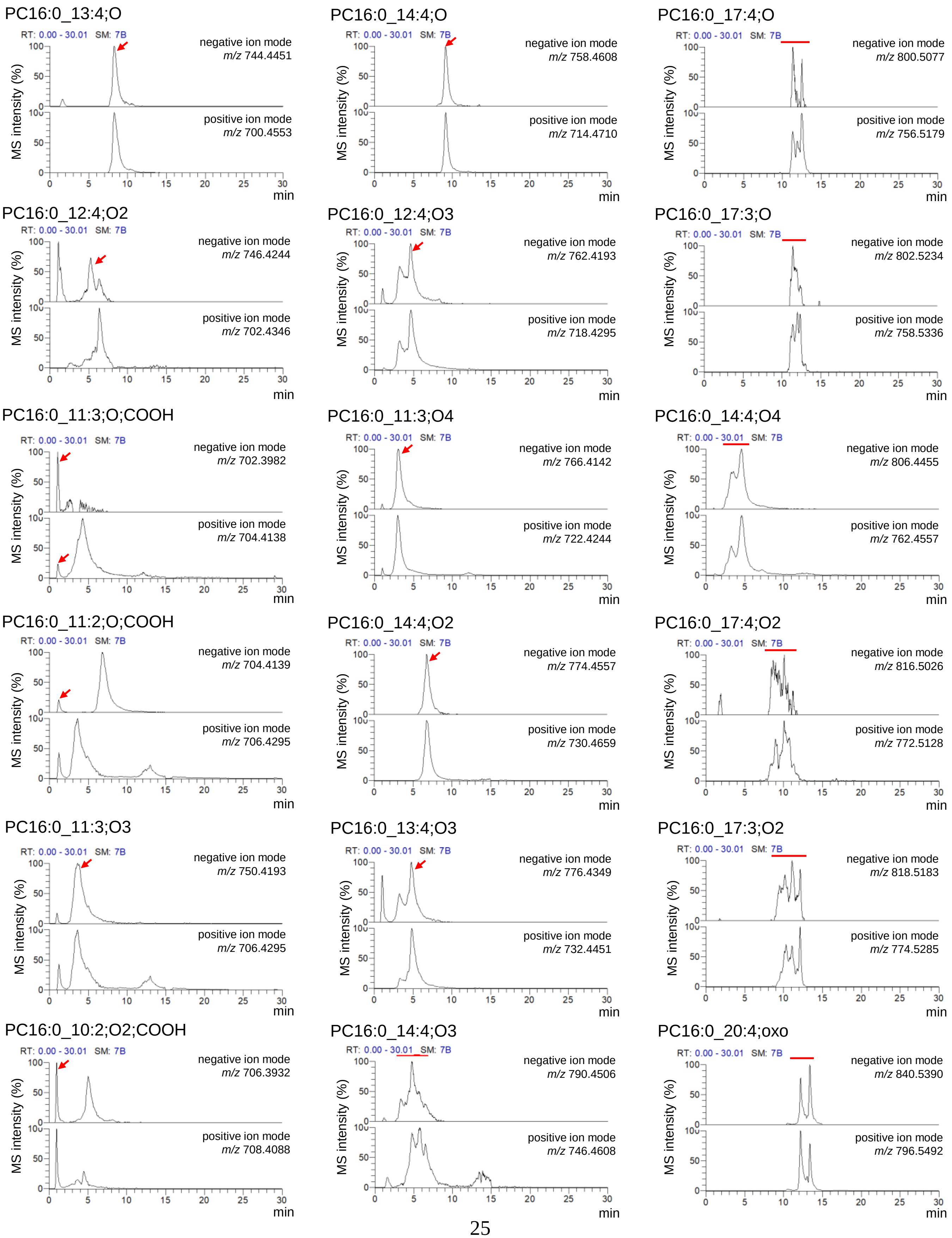

Continued Supplementary Figure 19

b

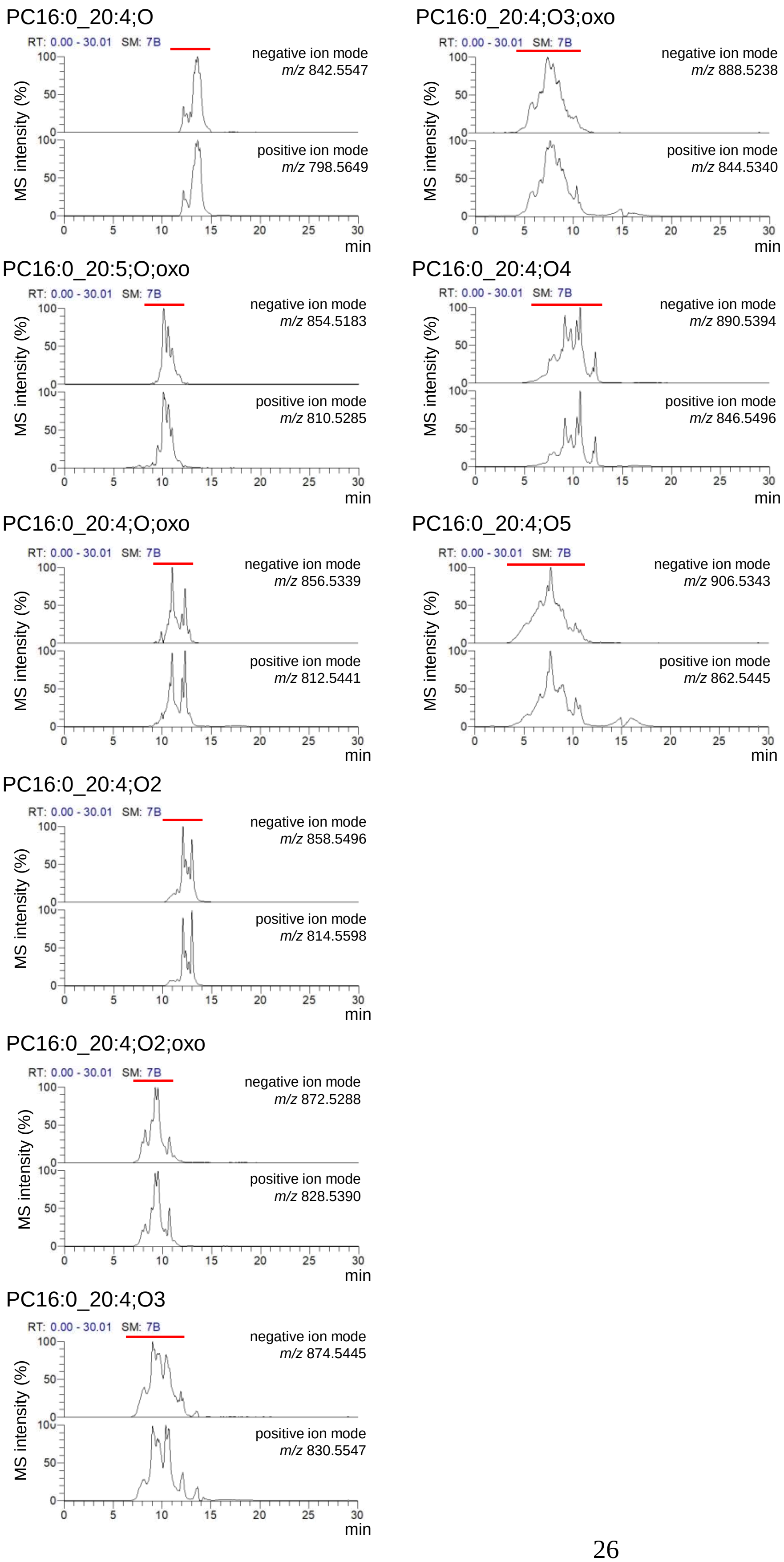

c

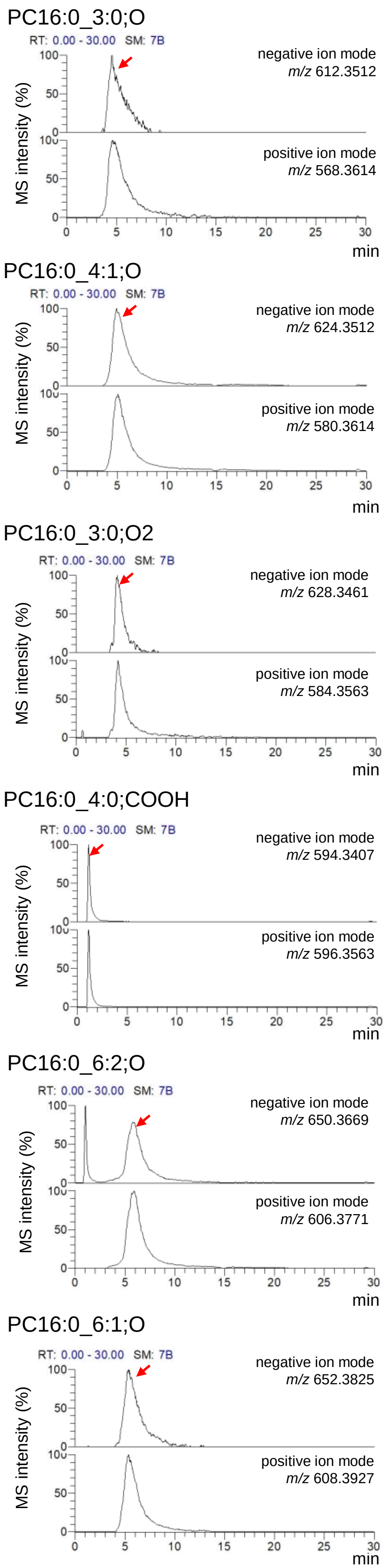

Continued Supplementary Figure 19

c

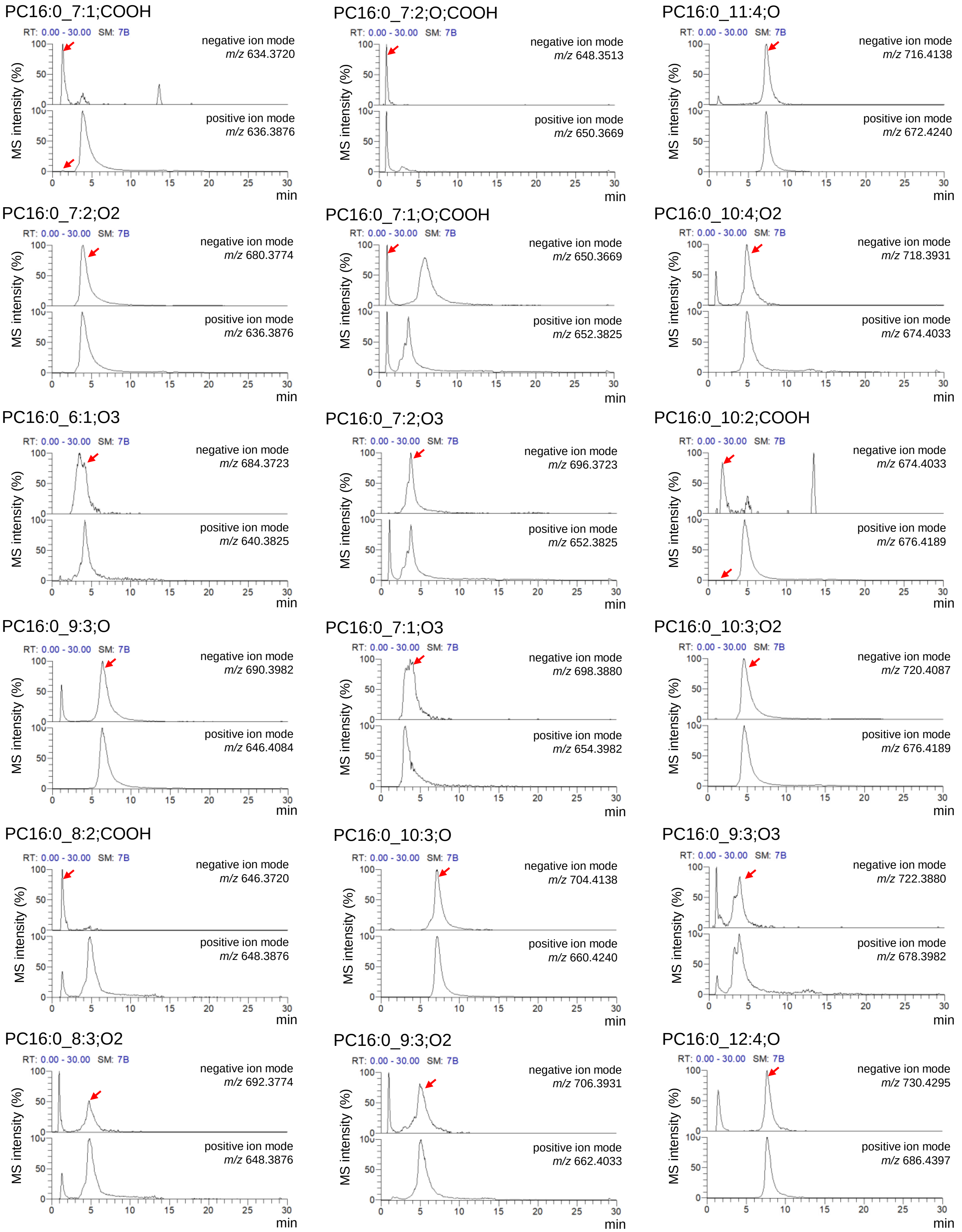

Continued Supplementary Figure 19

c

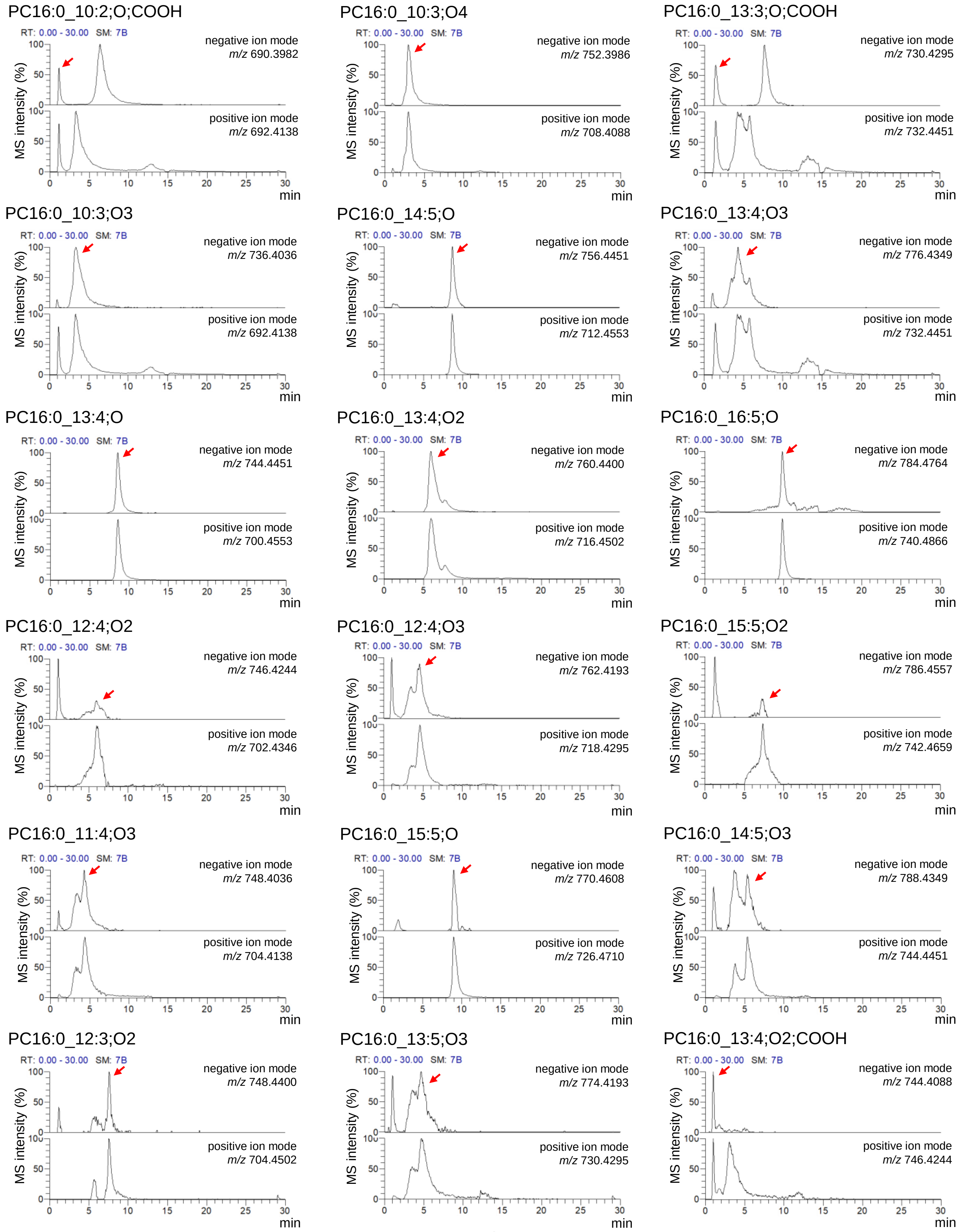

Continued Supplementary Figure 19

c

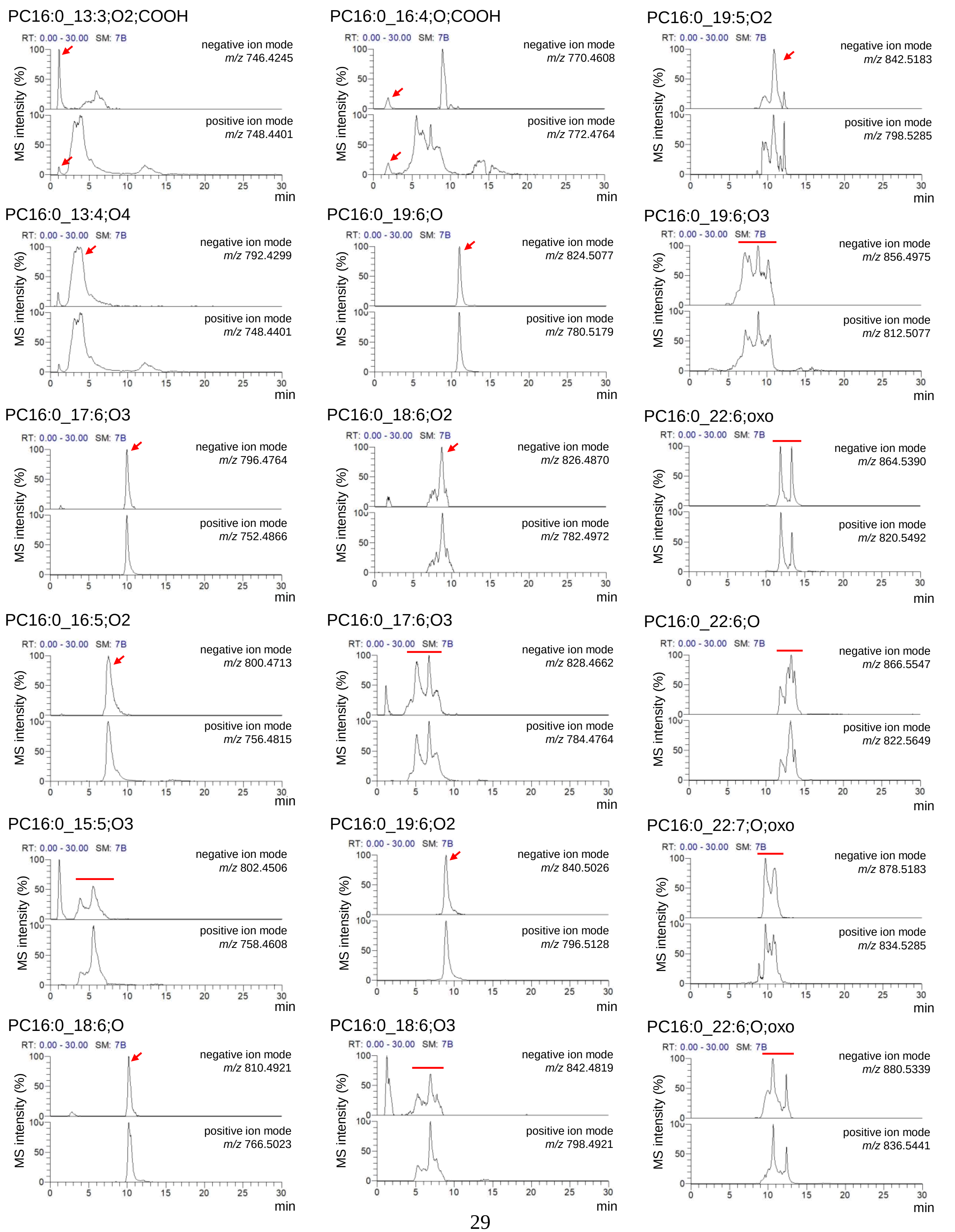

Continued Supplementary Figure 19

c

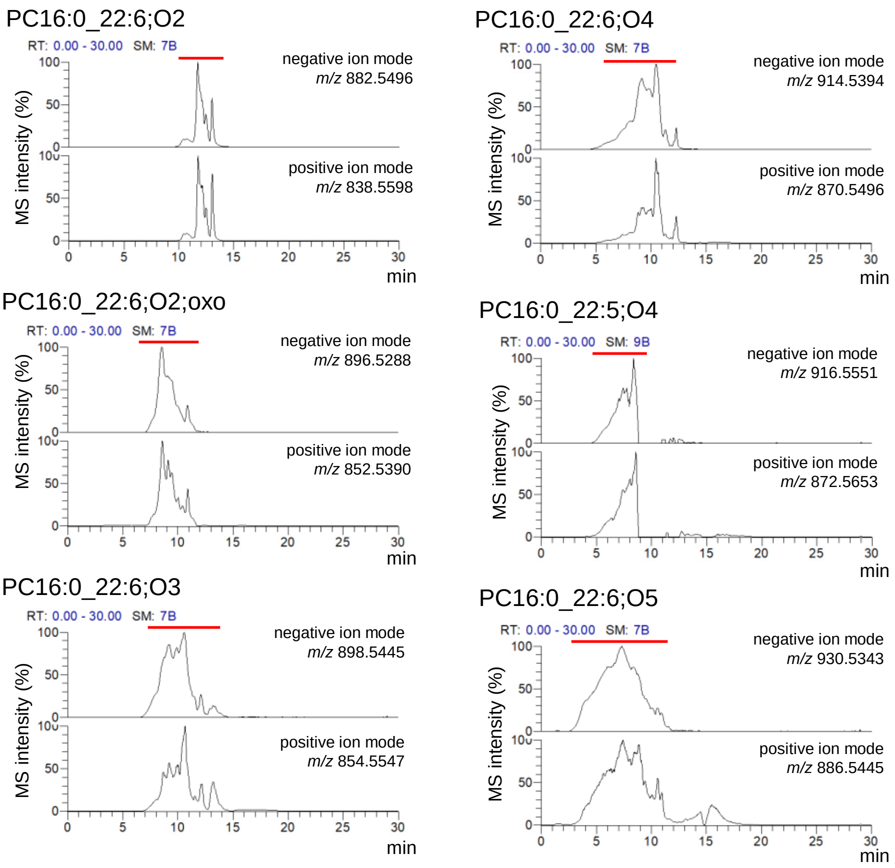

Supplementary Figure 19. EICs of individual oxPCs derived from PC16:0/18:2 (a), PC16:0/20:4 (b), and PC16:0/22:6 (c) measured by both negative and positive ion modes.

Supplementary References

1. Nishiyama, K. et al. Ibudilast attenuates doxorubicin-induced cytotoxicity by suppressing formation of TRPC3 channel and NADPH oxidase 2 protein complexes. *Br. J. Pharmacol.* **176**, 3723-3738 (2019).
