# Supplementary material for: Structural library and visualization of endogenously oxidized phosphatidylcholines using mass spectrometry-based techniques
Source: Nat Commun. 2021 Nov 3;12:6339. doi: 10.1038/s41467-021-26633-w (PMC8566498; doi:10.1038/s41467-021-26633-w)
Supplement: Supplementary file 3 — Description of Additional Supplementary Files [file 41467_2021_26633_MOESM3_ESM.docx]

**Description of Additional Supplementary Files**

File Name: Supplementary Data 1
Description: Oxidized PC16:0/PUFAs (18:2, 20:4, and 22:6) generated through either AAPH- or AAPH/hemin-induced LPO. Yellow; unreported oxPCs before our experiments.

File Name: Supplementary Data 2
Description: List of previous studies focusing on the structural analysis of oxPCs.

File Name: Supplementary Data 3
Description: MS/MS library for oxidized PCs.

File Name: Supplementary Data 4
Description: Limits of detection (LOD) and limits of quantitation (LOQ) of two standard oxPCs measured by LC/HRMS in the positive ion mode.
