# Supplementary material for: Structural library and visualization of endogenously oxidized phosphatidylcholines using mass spectrometry-based techniques
Source: Nat Commun. 2021 Nov 3;12:6339. doi: 10.1038/s41467-021-26633-w (PMC8566498; doi:10.1038/s41467-021-26633-w)
Supplement: Supplementary file 8 — Reporting Summary [file 41467_2021_26633_MOESM8_ESM.pdf]

nature research

Corresponding author(s): Ken-ichi Yamada

Last updated by author(s): Oct 1, 2021

Reporting Summary

Nature Research wishes to improve the reproducibility of the work that we publish. This form provides structure for consistency and transparency in reporting. For further information on Nature Research policies, see our Editorial Policies and the Editorial Policy Checklist.

Statistics

For all statistical analyses, confirm that the following items are present in the figure legend, table legend, main text, or Methods section.

n/a	Confirmed	
☐	☒	The exact sample size (n) for each experimental group/condition, given as a discrete number and unit of measurement
☐	☒	A statement on whether measurements were taken from distinct samples or whether the same sample was measured repeatedly
☐	☒	The statistical test(s) used AND whether they are one- or two-sided<br>*Only common tests should be described solely by name; describe more complex techniques in the Methods section.*
☒	☐	A description of all covariates tested
☒	☐	A description of any assumptions or corrections, such as tests of normality and adjustment for multiple comparisons
☐	☒	A full description of the statistical parameters including central tendency (e.g. means) or other basic estimates (e.g. regression coefficient) AND variation (e.g. standard deviation) or associated estimates of uncertainty (e.g. confidence intervals)
☐	☒	For null hypothesis testing, the test statistic (e.g. F, t, r) with confidence intervals, effect sizes, degrees of freedom and P value noted<br>*Give P values as exact values whenever suitable.*
☒	☐	For Bayesian analysis, information on the choice of priors and Markov chain Monte Carlo settings
☐	☒	For hierarchical and complex designs, identification of the appropriate level for tests and full reporting of outcomes
☒	☐	Estimates of effect sizes (e.g. Cohen's d, Pearson's r), indicating how they were calculated

Our web collection on statistics for biologists contains articles on many of the points above.

Software and code

Policy information about availability of computer code

Data collection	1. Nexera LC system (Shimadzu Co., Kyoto, Japan).<br>2. High-performance benchtop quadrupole Orbitrap mass spectrometer (Q Exactive, Thermo Fisher Scientific)<br>3. Xcalibur 4.2.47 software (Thermo Fisher Scientific).<br>4. MALDI linear ion trap mass spectrometer (MALDI LTQ XL; Thermo Fisher Scientific).<br>5. ImageQuest v. 1.0.1 software (Thermo Fisher Scientific).<br>6. Microsoft PowerPoint for Microsoft 365 (Microsoft).
Data analysis	1. Compound Discoverer 3.1 software (Thermo Fisher Scientific)<br>2. Xcalibur 4.2.47 software (Thermo Fisher Scientific).<br>3. ImageQuest v. 1.0.1 software (Thermo Fisher Scientific).<br>4. GraphPad Prism v. 9.2.0 (GraphPad Software).<br>5. Microsoft PowerPoint for Microsoft 365 (Microsoft).<br>6. MetaboAnalyst 4.0 (https://www.metaboanalyst.ca/).

For manuscripts utilizing custom algorithms or software that are central to the research but not yet described in published literature, software must be made available to editors and reviewers. We strongly encourage code deposition in a community repository (e.g. GitHub). See the Nature Research guidelines for submitting code & software for further information.

Data

Policy information about availability of data

All manuscripts must include a data availability statement. This statement should provide the following information, where applicable:

- Accession codes, unique identifiers, or web links for publicly available datasets
- A list of figures that have associated raw data
- A description of any restrictions on data availability

Data Availability: All data generated in this study are provided either in the main figures, supplementary data or source data file. Also, LC/HRMS(/MS) data have been deposited in Metabolomics Workbench under accession code PR001156[http://dev.metabolomicsworkbench.org:22222/data/DRCCMetadata.php?Mode=Project&ProjectID=PR001156&Access=DetP2954]. Source data are provided with this paper.

Field-specific reporting

Please select the one below that is the best fit for your research. If you are not sure, read the appropriate sections before making your selection.

☒ Life sciences ☐ Behavioural & social sciences ☐ Ecological, evolutionary & environmental sciences

For a reference copy of the document with all sections, see nature.com/documents/nr-reporting-summary-flat.pdf

Life sciences study design

All studies must disclose on these points even when the disclosure is negative.

Sample size	No statistical methods were used to predetermine sample size, but our sample sizes are similar to those reported in previous publications (Yamada KI. et al. Nat. Chem. Biol. 2016; 12(8):608-13). Sample sizes are indicated in the legends of each figure.
Data exclusions	No data were excluded from the analysis.
Replication	All attempts at replication of experimental findings were successful. All experiments were performed at least in triplicates.
Randomization	All samples and animals were randomly allocated into experimental groups.
Blinding	Blinding was not performed during data collection as knowledge of the treatment groups was required.

Reporting for specific materials, systems and methods

We require information from authors about some types of materials, experimental systems and methods used in many studies. Here, indicate whether each material, system or method listed is relevant to your study. If you are not sure if a list item applies to your research, read the appropriate section before selecting a response.

Materials & experimental systems

n/a	Involved in the study
☐	☒ Antibodies
☐	☒ Eukaryotic cell lines
☒	☐ Palaeontology and archaeology
☐	☒ Animals and other organisms
☒	☐ Human research participants
☒	☐ Clinical data
☒	☐ Dual use research of concern

Methods

n/a	Involved in the study
☒	☐ ChIP-seq
☒	☐ Flow cytometry
☒	☐ MRI-based neuroimaging

Antibodies

Antibodies used	Primary antibody: anti-CYP2E1 antibody, Atlas Antibodies, HPA009128, diluton 1:200<br>Secondary antibody: SignalStain Boost IHC Detection reagent (HRP, rabbit), Cell signaling, 8125, diluton 1:500
Validation	All antibodies used are of commercial origin and have been extensively used elsewhere.<br>anti-CYP2E1 antibody, Atlas Antibodies, HPA009128, https://www.atlasantibodies.com/products/antibodies/primary-antibodies/triple-a-polyclonals/cyp2e1-antibody-hpa009128/<br>SignalStain Boost IHC Detection reagent (HRP, rabbit), Cell Signaling Technology, 8125, https://www.cellsignal.jp/products/secondary-antibodies/boost-ihc-detection-reagent-hrp-mouse/8125

Eukaryotic cell lines

Policy information about cell lines

Cell line source(s)	RAW 264.7 (Cat# TIB-71, RRID:CVCL_0493) was obtained from the American Type Culture Collection (ATCC).
Authentication	Cell line identifies were confirmed by the commercial source (https://www.atcc.org/products/tib-71) and maintained separately and isolated from one another to avoid cross-contamination. Cell line was authenticated by STR method.
Mycoplasma contamination	All cell lines were tested negatively for mycoplasma contamination.
Commonly misidentified lines (See ICLAC register)	No commonly misidentified lines were used in this study.

Animals and other organisms

Policy information about studies involving animals; ARRIVE guidelines recommended for reporting animal research

Laboratory animals	Mice: C57BL6/J wild type, male, 8 weeks of age (Clea Japan, Inc.). Male C57BL6/J mice were housed in a light-controlled room (light/dark cycle of 12h/12h) at 24 ± 1 °C and 60% ± 10% humidity, and the animals had free access to water and CLEA Rodent Diet CE-2 (Clea Japan, Inc.).
Wild animals	This study didn't involve wild animals.
Field-collected samples	This study didn't involve sample collected from field.
Ethics oversight	All animal experimental procedures and animal care were approved by the Committee on Ethics of Animal Experiments, Graduate School of Pharmaceutical Sciences (Kyushu University) and Keio University School of Medicine and were conducted according to the Guidelines for Animal Experiments of the Graduate School of Pharmaceutical Sciences, Kyushu University and Keio University School of Medicine.

Note that full information on the approval of the study protocol must also be provided in the manuscript.
